# Data element investment strategy: How can leaders and followers innovate in dynamic market environments

**DOI:** 10.1371/journal.pone.0309659

**Published:** 2024-08-30

**Authors:** Yinhai Fang, Wei Wei, Rengang Su

**Affiliations:** 1 College of Economics and Management, Nanjing Forestry University, Nanjing, China; 2 College of Economics and Management, Nanjing University of Aeronautics and Astronautics, Nanjing, China; Shandong University of Science and Technology, CHINA

## Abstract

In the era of the digital economy, the data element investment strategy decisions and game mechanisms of leaders and followers are crucial issues to be studied. To explore the environment in which digital collaboration between enterprises benefits both parties, this study initially proposes a three-stage game model of leaders and followers based on the sequential game method. Subsequently, we analyze the investment strategy choices for leading and following enterprises across six scenarios within dynamic market environments. Finally, numerical simulations are employed to examine the effect of both strategies on the industry and society as a whole. The simulation shows that (1) The cooperation strategy is a more effective approach for enhancing data-driven innovation performance, but when it comes to mature markets, this strategy may conflict with the interests of followers. (2) Followers can benefit from the cooperation strategy by significantly boosting the growth rate of data elements, but it may cause enterprises to lose their original market scale. (3) Excessively high initial production costs can negatively affect the innovative performance of the industry and social wealth, whereas mature industries can achieve greater industry performance and social welfare through investment in data elements. Considering the environmental characteristics of the digital economy, the findings of this study elucidate the ramifications of innovation strategies on enterprises, industries, and society, providing positive insights for two types of enterprises with different strengths to make apt decisions regarding digital cooperation.

## 1. Introduction

**Fig 1:**
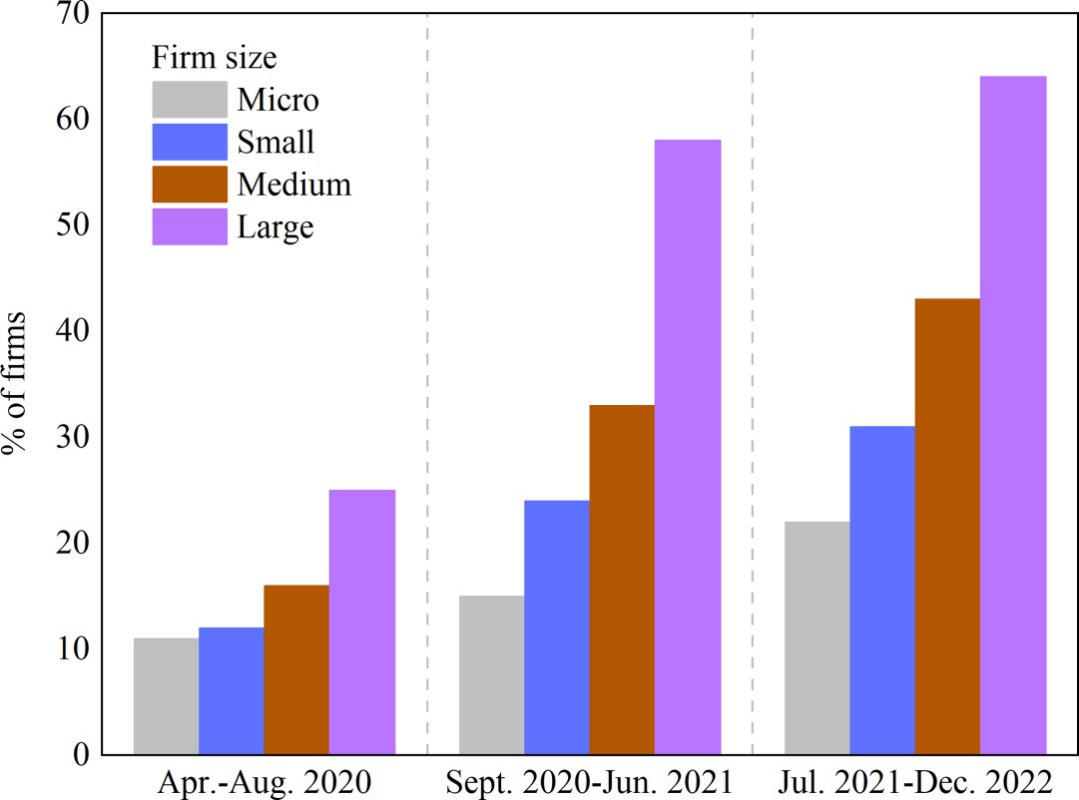
Share of firms investing in digital solutions by firm size.

In the context of the digital transformation era, businesses seek to enhance their industrial performance by collecting and analyzing data throughout the product life cycle [1, 2]. The analysis of data elements has the potential to facilitate economic transformation, particularly in the reshaping of product and factor markets, which offers new opportunities for enterprise innovation [3, 4]. In practice, the National Center for Supercomputing Applications (NCSA) has analyzed data elements to reveal obscure market patterns, cash flow trends, and customer preferences, which enhances global competitiveness across various industries such as agriculture [5], healthcare [6], energy [7], and finance [8]. Companies such as Google, Amazon, Alibaba, and Twitter have greatly benefited from digital technology and have developed various strategies employing data elements to counter competitors [9]. Currently, a multitude of enterprises have initiated digital innovation by integrating consumer knowledge and information, thereby developing systematic frameworks for strategic management [10]. As illustrated in Fig 1 of the Digital Progress and Trends Report 2023, released by the World Bank Group, the proportion of firms of varying sizes investing in digital solutions is on the rise. Nevertheless, the rate of digitization differs considerably between enterprises, resulting in an increasing disparity in digital capabilities. Larger organizations are making more substantial investments in digital technologies, whereas less than half of small and medium-sized enterprises have adopted digital transformation. Due to limited resources, many enterprises encounter difficulties in engaging in digital transformation, which hinders their efforts to develop and innovate, ultimately impacting their competitiveness. In general, the degree of enterprise digitization remains relatively low, indicating that the challenge of embracing digitization is significant and requires immediate attention [11, 12].

Data elements are regarded as the basis for enterprise innovation in unstable markets, encompassing knowledge management, decision support, business processes, and supply chain management [13, 14]. In the context of the post-pandemic era, the increasingly volatile market environment represents a significant factor that enterprises must consider when executing their data element investment strategies. The intensification of cross-border competition, the rapid pace of scientific and technological change, the saturation of markets, and the homogenization of products are all factors that contribute to an environment of increasing uncertainty for companies, which necessitates a heightened focus on the changing environment. This is because the efficacy of digital strategy is contingent upon the enterprise’s digital process and the dynamic environment of the match. Failure to align these two factors increases the likelihood of the digital paradox, whereby the digital transformation is expected to bring more opportunities and convenience, yet the actual outcome is minimal. To cope with the drastic changes in the dynamic environment and avoid falling into the dilemma of the digital paradox, some companies now choose to implement digital co-innovation strategies with their competitors [15, 16]. For instance, BMW and Daimler launched a partnership to expand their digital business in 2018, catering to the field of innovative mobility services. In 2021, Walmart and JD.com initiated a collaborative project to enhance the identification and operation of VIP users through the application of digital technologies. Although these companies are amenable to collaboration and exchange of digital technologies, they still compete with each other within business domains. However, the implementation of a cooperation strategy may potentially give rise to certain risks, such as fair competition or trade secrets disclosure, resulting in increasing data conflicts between enterprises. These conflicts are particularly evident between two types of enterprises with different market positions: leading and following enterprises [17]. Leaders are the dominant players in an industry, and they possess more resources and a greater ability to influence the industry, while followers have relatively weak resource bases and low market responsiveness, and tend to compensate for these disadvantages through learning strategies [18]. These two types of enterprises are typical subjects in digital innovation activities, and their strategic choices and innovation capabilities are constrained by different characteristics and behaviors.

Although previous studies have revealed the selections of enterprise strategy under environmental uncertainty [19, 20], there remains a lack of in-depth and comprehensive understanding of the data element investment strategies adopted by leading and following enterprises in a dynamic market environment with significant digital economy characteristics. Based on the above discussion, this paper employs the sequential game method to illustrate the significance of market power asymmetry and to simulate the performance of cooperative and independent strategies in a variety of dynamic market environments. Consequently, this paper primarily aims to support enterprises in their investment decisions on data elements, especially by solving two unsolved problems, namely: What are the game mechanisms of leaders and followers in the market? What are the characteristics of leaders’ and followers’ willingness to cooperate and their game strategy selections in different market environments, with a particular focus on their impact on overall industry innovation performance, consumer surplus, and social wealth? In general, this paper constructs a three-phase game model between leaders and followers in digital-driven innovation. Additionally, we uncover the game mechanisms employed by enterprises when investing in data elements in six scenarios of dynamic market environments. Finally, we assess the effects of enterprises’ data element investment on industry and society under varying circumstances.

## 2. Literature review

### 2.1. Game theory in enterprise management

Game theory is the study of mathematical models that analyze the choice of the best strategy in situations where there is a potential conflict between rational decision-makers who interact under one or more conditions [21]. The method has wide applicability as it can assist enterprises in developing a clearer decision-making framework, improving strategic planning, and increasing the possibility of inter-subject cooperation [22]. Thus, game models are widely used in the field of management to solve business-related problems, such as strategic decision-making [23, 24], resource allocation [25], and risk assessment [26].

The asymmetric relationship between firms in the market is the norm, which implies that the subject’s decision-making process is sequential. The sequential game is a typical dynamic game in which the subject’s decision-making occurs in a time sequence. The latter actor responds after observing the decision-making of the first actor, and the Stackelberg model is designed to reflect this asymmetric competition [27, 28]. Amir et al. examined the potential advantages of employing sequential games between firms in a non-cooperative environment at the R&D stage, where the leader is typically the firm with greater absorptive capacity [29]. Sinha et al. used a two-layer evolutionary algorithm with a nested structure to solve a multi-leader-follower competition problem and found that the leader benefits from a significant first-mover advantage in the competition, while the followers’ objective function values do not necessarily exhibit a consistent improvement [30]. Küçükaydın et al. illustrated how new market entrants can optimize their profits by determining the location and desirability of new facilities in the context of existing competitors [31]. Thus, the game model is a systematic and quantitative tool that can be used to describe the competitive relationship between leading and following firms [32]. Furthermore, the method is effective in revealing the characteristics of the industry environment, which permits a clear and systematic presentation of the economic phenomena and the logic of competition in a given industry. Xin and Sun proposed a differential oligopoly game model that investigates how firms can cope with water scarcity by optimizing their production plans and water conservation strategies in the presence of sticky product prices and water right prices [33]. Xiao and Cui used a multi-stage evolutionary model concerned with exploring the impact of factors such as technological improvements on the strategic choices of shipping firms and how government carbon quota policies adjust to changes in demand in the shipping market during low and high seasons [34]. In summary, the game model, as an important tool for analyzing the decision-making behavior of economic agents, shows unique advantages in interpreting the complex relationship between enterprises with different market positions. The incorporation of the game model into the realm of firm decision-making enables a more comprehensive analysis of the costs and benefits, particularly in contexts that are characterized by complexity and uncertainty.

### 2.2. Data elements and digital innovation in enterprise management

Data elements are the key drivers of the digital economy [35], which provide valuable insights for businesses, including market analysis, trend forecasting, operational efficiency improvement, risk management, new product development, and reinforcement of competitive advantages.

Scholars generally agree that data elements in business development are extensive, effective, and worthwhile. Data elements can improve the efficiency of business operations and innovation [36], while innovation sustainability contributes to identifying market opportunities, predicting customer needs, and analyzing customer purchasing decisions [37]. Enterprises located at the center of social networks have advantages in the acquisition and processing of data elements based on the techno-economic paradigm of the Internet, and the real-time analysis of data from different sources creates new opportunities for business development [38]. Data-driven innovation practices can facilitate enterprises’ rapid decision-making processes in today’s volatile markets [9, 39] and subsequently innovate business models [40]. Enterprises in the supply chain can use digital assets to create precise and timely strategic plans during operational processes, resulting in benefits from digitalization growth [41, 42]. Consequently, the innovation performance that enterprises can achieve is based on their data elements.

Enterprise management relies on data to inform decision-making processes, thereby emphasizing the imperative of investing in data elements to obtain valuable information. Despite the increasing interest in technological advancements and data analytics, many organizations encounter challenges in utilizing data elements. The acceleration of globalization and the rapid application of new technologies have led to an intensification of competition in various industries. Concurrently, the intensification of global economic uncertainty has rendered the market environment increasingly complex [43]. In addition, the processing of data elements in complex environments is a time-consuming and computationally complex task [44]. When data elements are not processed quickly and effectively to support enterprise decisions, their value is reduced [45]. As a result, enterprises are compelled to address the fundamental question of which environment is optimal for the implementation of a digital collaboration strategy.

In conclusion, enterprises should collaborate and innovate to collectively overcome the challenges posed by the dynamic market environment. The primary objectives for scholars in the fields of digital innovation and strategic management are investing in data elements within fiercely competitive marketplaces, selecting digital strategies that align with the quality of business, and ultimately contributing to the enhancement of innovation performance. Data-driven innovation extends beyond product innovation, as enterprises increasingly use information networks and data elements to revolutionize their business models [46] and service models [1]. This highlights the importance and urgency of exploring investment strategies for data elements to promote innovation within the enterprise.

### 2.3. Enterprise management considering data element investment

The development of digital technology has emerged as a pivotal catalyst for enterprise innovation, with the many studies suggesting that investment in data elements can yield benefits. Some scholars employed case studies to illustrate the value of data investment strategies on enterprise development. These advantages are reflected in the establishment of a robust data information system, which enables a more nuanced understanding of market dynamics and customer demand. Furthermore, it facilitates the optimization of production efficiency and organizational structure, thereby enhancing innovation and business value [47, 48]. Other scholars used empirical analyses to examine the effect of data element investment. Their findings suggest that enterprises can enhance human capital, enterprise performance, and customer expectations when investing in data technology, which deepens the organization’s understanding of how to use its data resources [49–52]. Currently, the existing studies use game theory to examine the investment of enterprise data elements, with a main focus on supply chain management. This approach offers insights into the selection of optimal strategies for reducing the costs associated with data element investment to obtain innovation performance, thereby maximizing the value of data [53, 54].

In general, it is widely acknowledged among scholars that data element investment plays a pivotal role in enterprise development. However, there is a paucity of research examining the strategic decision-making processes of enterprises operating in dynamic market environments. Furthermore, based on the sequential game and simulation analysis, we examine two perspectives concurrently: the sequential yield decision of enterprises and the investment decision with the objective of overall profit maximization, which elucidates the disparate market power positions of leaders and followers. Besides, data element investment is confronted with a complex and evolving environment, and there are few studies on the resistance from the environment that enterprises encounter when engaging in digital collaboration. The three-stage game model we develop reasonably introduces the factors that may have a significant impact on enterprises’ decisions in digital economy scenarios, with a particular focus on the choice of enterprises’ data element investment strategies under conditions of environmental uncertainty.

## 3. Game model

**Fig 2:**
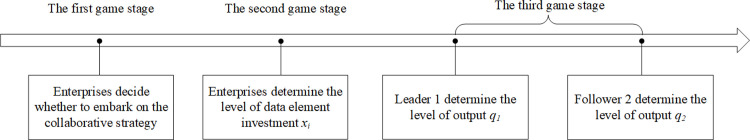
The sequence of events.

In light of the preceding review of data element investment models, we assess that the Stackelberg model offers a perspicuous depiction of the market structure [55], which allows for a comparison of the outputs and profits under cooperative and independent strategies. This paper examines the innovation process of firms driven by data elements and models this process with economic factors [56, 57], technological factors [58], and market demand factors [59, 60] in the digital economy environment. Especially, the model involves three stages of decision-making, as shown in Fig 2. Firstly, the enterprise determines whether to establish a cooperation strategy with another enterprise. Secondly, the two enterprises concurrently determine their respective investment levels of data elements. Finally, the two enterprises engage in the Stackelberg game. Without loss of generality, we assume that enterprise 1 is the leader and determine the output *q*_1_ first in the game. Enterprise 2 is the follower, which determines its output *q*_2_ after observing *q*_1_ Based on the decision-making characteristics of the three stages discussed, this paper presents a three-stage game model for data element investment in enterprise collaborative innovation.

### 3.1. Basic description of the model

To simplify the model analysis process, we assume that there are two enterprises *i* and *j* producing products in the market. The amount that enterprise’s output is denoted as *q*, and the total output *Q* = *q*_*i*_ + *q*_*j*_. To determine the product price of enterprise *i*, we utilize the inverse demand function *p*_*i*_ (t) = Φ (*x*_i_) ‒ *Q*, where Φ (x_i_) represents the market size of enterprise *i*, and *x*_*i*_ (*x*_*i*_∈(0,1)) represents the data element investment level of enterprise *i*. The enterprise *i*’ market size function Φ_*i*_ is given as follows, where Φ_0_ represents initial market size.


$$\Phi_{i}=\Phi_{0}\times \left(1+x_{i}\right.)$$ (1)

The value of digital assets is subject to the quality of data processing experience and hardware facilities, and with the strong positive externality of data element investment in consideration [61]. Here, the digital assets growth rate *g* of enterprises *i* and *j* are assumed to meet the following conditions. Where λ is the spillover effect, represents the marginal cross-effect of enterprise *j’s* digital assets on enterprise *i*, μ denotes the elasticity of substitution between the two, represents the extent to which follower’s digitized research results are replaceable by the leader, and *g*_0_ is the steady-state growth rate of economy. Especially, the digital assets growth rate encompasses not only the intrinsic benefit derived from the level of data element investments made by the firm itself, but is additionally influenced by spillover coefficients and elasticity of substitution acting on the level of data element investments made by the two firms.


$$\begin{array}{l} g_{0}x_{i}+g_{0}\mu \lambda x_{i}+g_{0}(1-\mu )\lambda x_{j}=g_{i}x_{i}\\ g_{0}x_{j}+g_{0}\mu \lambda x_{i}+g_{0}(1-\mu )\lambda x_{j}=g_{j}x_{j} \end{array}$$ (2)

Then, the next step is to calculate the marginal production cost of enterprise *i*. Data element investment can decrease financing and management costs. Generally, we assume enterprises *i* and *j* have zero fixed production costs. Therefore, the formula is as follows:

$$c_{i}=c_{0}-\left(1+g_{i}\right)*x_{i}$$ (3)

Where *c*_0_ denotes initial marginal production cost, and (1 + *g*_*i*_) * *x*_*i*_ represents the digital assets of enterprise *i*. In addition, we assume that the cost of capitalizing data element of enterprise *i* is $\frac{\gamma }{2}x_{i}^{2}(\gamma >0)$, where γ is the cost coefficient of digital assets. The capitalization efficiency of data element decreases as γ increases. Taking into account the cost of redundant information, the cost of capitalizing data elements is characterized by diminishing marginal returns.

Then, the innovation performance of enterprise *i* can be calculated as the difference between sales revenue and the total cost with investing for data elements:

$$\pi_{i}=\left[\Phi_{i}-q_{i}-q_{j}\right]\times q_{i}-\left[c_{0}-\left(1+g_{i}\right)*x_{i}\right]\times q_{i}-\frac{\gamma }{2}x_{i}^{2}$$ (4)

This paper not only focuses on the game behavior between enterprises, but also examines the equilibrium situation that benefits society as a whole. Therefore, in the context of equilibrium *S*^*m*^, the total social welfare *W*^*m*^ is defined as the sum of the total innovation performance of two enterprises *R*^*m*^ and the consumer surplus *V*^*m*^.


$$W^{m}=R^{m}+V^{m}$$ (5)

$$V^{m}=\frac{1}{2}{\left(q_{i}^{m}+q_{j}^{m}\right)}^{2}$$ (6)

Where $q_{i}^{m}$ and $q_{j}^{m}$ are the respective outputs of enterprise *i* and *j* in scenario *S*^*m*^.

**Fig 3:**
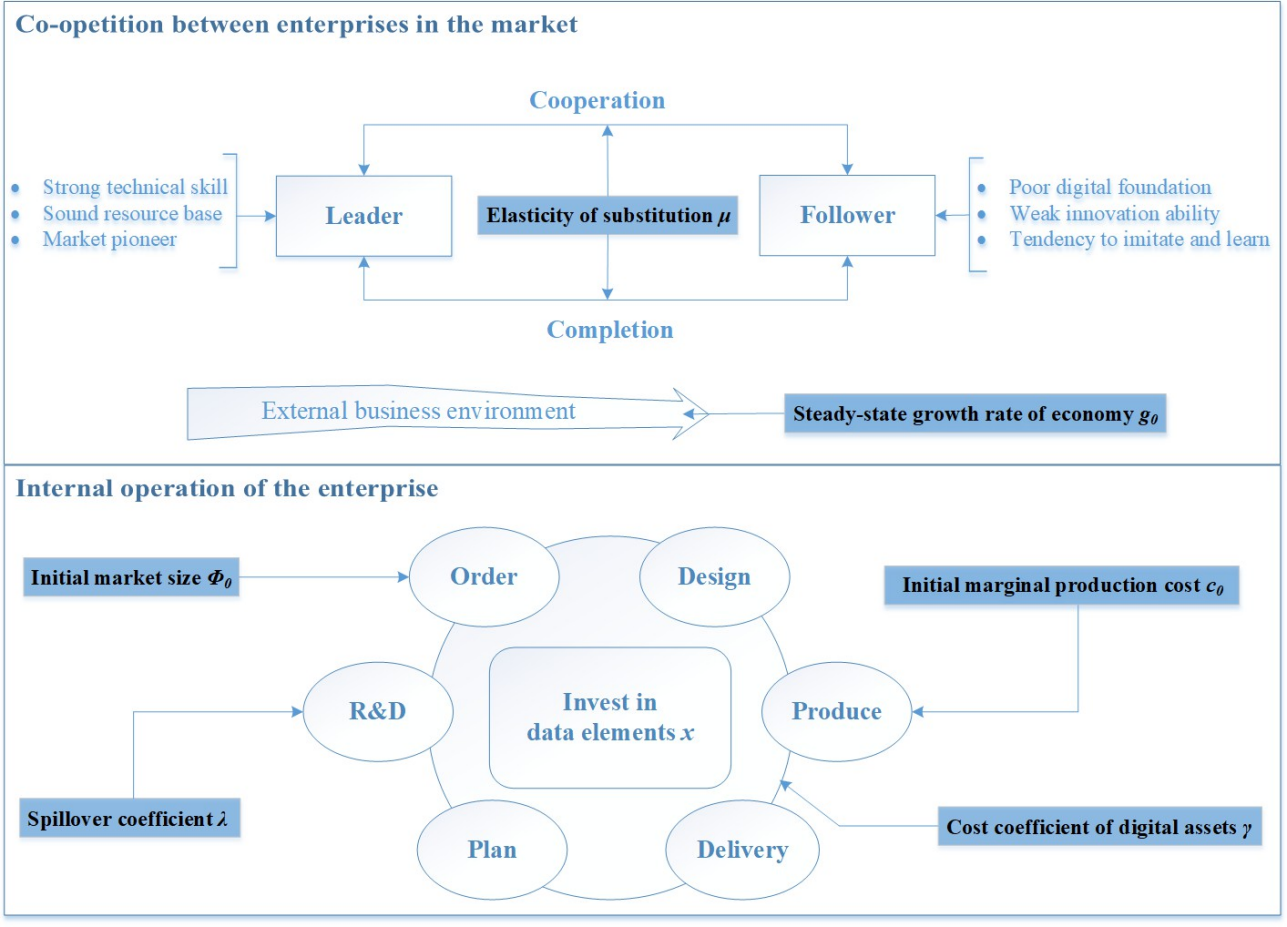
Dynamic market environment mechanism of action diagram.

Based on the above description of the model, we introduce the role of these six dynamic market environment parameters in inter-enterprise competition and enterprise operation management in detail, as shown in Fig 3. Especially when enterprises compete in the market, their products and services are affected by the elasticity of substitution. In addition, the steady-state growth rate of the economy, which represents the industry’s benchmark, transmits supply and demand information from the external environment. From the perspective of enterprise operation management, the initial market size, initial marginal production cost, and spillover coefficient influence the order, production, and R&D links, respectively. Meanwhile, the cost coefficient of digital assets affects the transformation process from production link to physical delivery.

### 3.2. Model solving

This paper applies the backward induction to solve the model. This method analyzes the decision-making strategies of behavior subjects from the last stage of the dynamic game. The analysis progresses recursively to the preceding stage of the game until its starting point. This paper assumes that there are two enterprises operating in the market, producing homogeneous products and services, namely leader 1 and follower 2. In particular, the two strategies are selected based on the principle of utility maximization. According to this principle, each enterprise chooses whether to cooperate with another enterprise to invest in data elements.

#### 3.2.1. Output decision during the third game stage

In the context of digital innovation, both parties should first make decisions about outputs and assess the possibility of carrying out the digital collaborative innovation strategy based on the analysis results of this stage. Firstly, according to Eq (2), we calculated the steady-state growth rate for the leader and follower, as given in Eq (7).


$$\begin{array}{l} g_{1}=g_{0}\left[1+\lambda \mu +(1-\mu )\lambda \frac{x_{2}}{x_{1}}\right]\\ g_{2}=g_{0}\left[1+\lambda \mu \frac{x_{1}}{x_{2}}+(1-\mu )\lambda \right] \end{array}$$ (7)

Secondly, the output of the follower is calculated by taking the first-order partial derivative of the profit function Eq (4) for *q*_2_ and then setting it equal to 0, as given in Eq (8).


$$q_{2}^{*}\left(q_{1}\right)=\frac{\Phi_{0}\left(1+x_{2}\right)-q_{1}-c_{2}}{2}$$ (8)

Thirdly, by substituting Eq (8) with the profit function of the leader, we can get the output of the leader, as given in Eq (9).


$$q_{1}^{*}=\left(-\frac{\Phi_{0}}{2}-\frac{1}{2}g_{0}\lambda \mu +\frac{1}{2}g_{0}\lambda -\frac{1}{2}g_{0}-\frac{1}{2}\right)x_{2}+\left(\Phi_{0}+\frac{1}{2}g_{0}\lambda \mu +g_{0}+1\right)x_{1}+\frac{\Phi_{0}-c_{0}}{2}$$ (9)

Substitute Eq (9) into Eq (8) to obtain the output of the follower, as given in Eq (10).


$$q_{2}^{*}=\left(\frac{3}{4}\Phi_{0}-\frac{1}{4}g_{0}\lambda \mu +\frac{1}{4}g_{0}\lambda +\frac{3}{4}+\frac{3}{4}g_{0}\right)x_{2}+\left(-\frac{1}{2}\Phi_{0}-\frac{1}{2}+\frac{1}{4}g_{0}\lambda \mu -\frac{1}{2}g_{0}\right)x_{1}+\frac{\Phi_{0}}{4}-\frac{c_{0}}{4}$$ (10)

Finally, we calculated the equilibrium profits of the leader and the follower, as given in Eqs (11–12).


$$\pi_{1}=\frac{1}{2}{\left[Ax_{2}+Bx_{1}+\frac{\Phi_{0}-{\text{c}}_{0}}{2}\right]}^{2}-\frac{\gamma }{2}x_{1}^{2}$$ (11)

$$\pi_{2}={\left[Cx_{2}+Dx_{1}+\frac{\Phi_{0}-{\text{c}}_{0}}{4}\right]}^{2}-\frac{\gamma }{2}x_{2}^{2}$$ (12)

Especially, $A=-\frac{\Phi_{0}}{2}-\frac{1}{2}g_{0}\lambda \mu +\frac{1}{2}g_{0}\lambda -\frac{1}{2}g_{0}-\frac{1}{2};$ $B=\Phi_{0}+\frac{1}{2}{\text{g}}_{0}\lambda \mu +{\text{g}}_{0}+1$; $C=\frac{3}{4}\Phi_{0}-\frac{1}{4}g_{0}\lambda \mu +\frac{1}{4}g_{0}\lambda +\frac{3}{4}+\frac{3}{4}g_{0}$; $D=-\frac{1}{2}\Phi_{0}-\frac{1}{2}+\frac{1}{4}{\text{g}}_{0}\lambda \mu -\frac{1}{2}{\text{g}}_{0}$.

#### 3.2.2. Data element investment decision during the second game stage

In this stage, the leader and the follower determine their investment levels for data elements simultaneously. Considering the differences in objective functions between independent and collaborative strategies of investing data elements, we address the above two cases separately.

First, when leader 1 and follower 2 invest data elements independently, their profit functions are derived from *x*_1_ and *x*_2_ respectively. Equations are then constructed to obtain the equilibrium solution ($x_{1}^{D},x_{2}^{D}$), as given in Eqs (13–14).


$$x_{1}^{D}=\frac{B\left(\Phi_{0}-{\text{c}}_{0}\right)\left(AC-2C^{2}+\gamma \right)}{\left(4B^{2}-4\gamma \right)C^{2}-4ABCD-2B^{2}\gamma +2\gamma^{2}}$$ (13)

$$x_{2}^{D}=\frac{C\left(\Phi_{0}-{\text{c}}_{0}\right)\left(B^{2}-2BD-\gamma \right)}{\left(-4C^{2}+2\gamma \right)B^{2}+4ABCD+4C^{2}\gamma -2\gamma^{2}}$$ (14)

By substituting Eqs (13–14) into Eqs (9–10) and Eqs (11–12), we obtain the equilibrium output sum $Q_{1}^{D}$ and $Q_{2}^{D}$, and the equilibrium profit sum $\pi_{1}^{D}$ and $\pi_{2}^{D}$ respectively when they invest data elements independently.

Second, when leader 1 and follower 2 invest data elements collaboratively, their shared objective is to maximize the overall industry innovation performance *R*^*C*^ as determined by Eqs (11–12), as given in Eq (15).


$$R^{C}=\frac{1}{2}{\left[Ax_{2}+Bx_{1}+\frac{\Phi_{0}-{\text{c}}_{0}}{2}\right]}^{2}-\frac{\gamma }{2}x_{1}^{2}+{\left[Cx_{2}+Dx_{1}+\frac{\Phi_{0}-{\text{c}}_{0}}{4}\right]}^{2}-\frac{\gamma }{2}x_{2}^{2}$$ (15)

The overall industry innovation performance function *R*_*C*_ comprises two sections for taking partial derivatives for *x*_1_ and, *x*_2_. Thus, a set of equations is built to determine the equilibrium solution ($x_{1}^{C},x_{2}^{C}$) for the data element investment levels of enterprises 1 and 2, as given in Eqs (16–17):

$$x_{1}^{C}=\frac{((B+D)\gamma -(A-2C)(AD-BC))\left(\Phi_{0}-{\text{c}}_{0}\right)}{2\gamma^{2}+\left(-2A^{2}-2B^{2}-4C^{2}-4D^{2}\right)\gamma +4{\left(AD-BC\right)}^{2}}$$ (16)

$$x_{2}^{C}=\frac{((A+C)\gamma +(B-2D)(AD-BC))\left(\Phi_{0}-{\text{c}}_{0}\right)}{2\gamma^{2}+\left(-2A^{2}-2B^{2}-4C^{2}-4D^{2}\right)\gamma +4{\left(AD-BC\right)}^{2}}$$ (17)

By substituting Eqs (16–17) into Eqs (9–10) and Eqs (11–12), leader 1 and follower 2 equilibrium output sum $Q_{1}^{C}$, $Q_{2}^{C}$ and equilibrium profit sum $\pi_{1}^{C}$, $\pi_{2}^{C}$ were obtained respectively.

The equilibrium output and profit of two enterprises under the two strategies can be obtained, as shown in Table 1.

**Table 1:** Equilibrium results.

	Invest data elements independently	Invest data elements collaboratively
Data element investment	$x_{1}^{D}$	$x_{1}^{c}$
	$x_{2}^{D}$	$x_{2}^{C}$
Equilibrium output	$Q_{1}^{D}=Ax_{2}^{D}+Bx_{1}^{D}+\frac{\Phi_{0}-{\text{c}}_{0}}{2}$	$Q_{1}^{C}=Ax_{2}^{C}+Bx_{1}^{C}+\frac{\Phi_{0}-{\text{c}}_{0}}{2}$
	$Q_{2}^{D}=Cx_{2}^{D}+Dx_{1}^{D}+\frac{\Phi_{0}}{4}-\frac{c_{0}}{4}$	$Q_{2}^{C}=Cx_{2}^{C}+Dx_{1}^{C}+\frac{\Phi_{0}}{4}-\frac{{\text{c}}_{0}}{4}$
Equilibrium profit	$\pi_{1}^{D}=\frac{1}{2}{\left[Ax_{2}^{D}+Bx_{1}^{D}+\frac{\Phi_{0}-{\text{c}}_{0}}{2}\right]}^{2}-\frac{\gamma }{2}\left(x_{1}^{D}\right.{)}^{2}$	$\pi_{1}^{C}=\frac{1}{2}{\left[Ax_{2}^{C}+Bx_{1}^{C}+\frac{\Phi_{0}-{\text{c}}_{0}}{2}\right]}^{2}-\frac{\gamma }{2}{\left(x_{1}^{C}\right)}^{2}$
	$\pi_{2}^{D}={\left[Cx_{2}^{D}+Dx_{1}^{D}+\frac{\Phi_{0}}{4}-\frac{{\text{c}}_{0}}{4}\right]}^{2}-\frac{\gamma }{2}{\left(x_{2}^{D}\right)}^{2}$	$\begin{array}{r} \pi_{2}^{C}={\left[Cx_{2}^{C}+Dx_{1}^{C}+\frac{\Phi_{0}}{4}-\frac{{\text{c}}_{0}}{4}\right]}^{2}\\ -\frac{\gamma }{2}\left(x_{2}^{C}\right.{)}^{2} \end{array}$

**Fig 4:**
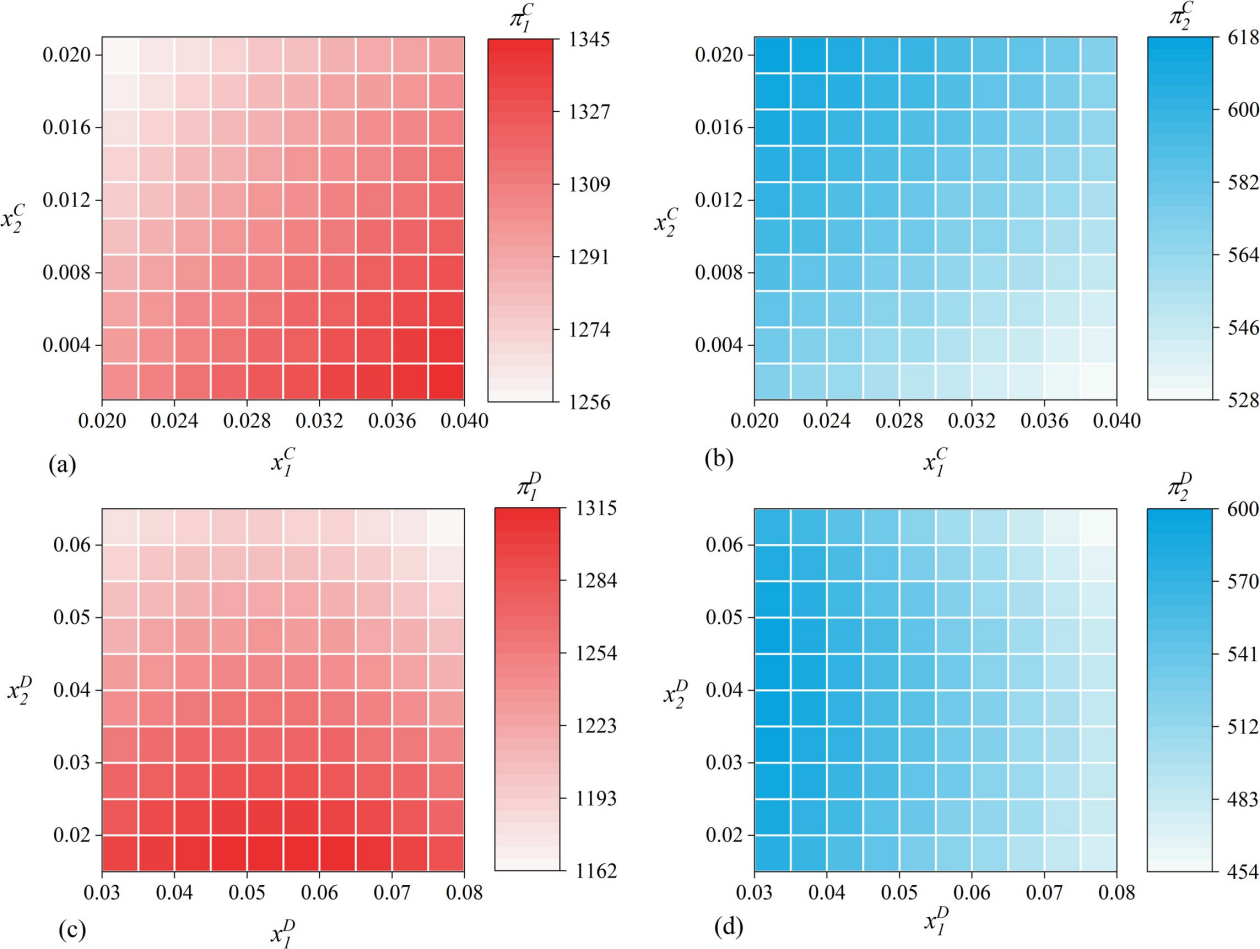
Heat map of the equilibrium profit gained from enterprise data element investment.

The equilibrium results presented in Table 1 are utilized to create heat maps that illustrate the profitability of the leader and follower’s data element investment strategies in the collaborative and independent contexts, respectively. Fig 4 illustrates that in the case of the collaborative strategy, both enterprises should invest as much as possible in data elements to achieve substantial profits. In contrast, in the case of the independent strategy, the peak value of the profits of both enterprises is achieved at the mid-range of the investment volume. However, the above analysis is based on the assumption of constant market parameters and ignores the dynamic characteristics of the digital economy environment. Consequently, we present a comprehensive analysis of six dynamic market environment characteristics, comparing the performance of leader and follower data element investment strategies in the following sections.

### 4. Data element investment strategies in dynamic market environments

In this study, the sequential game is used to establish the equilibrium output and profit functions of leader 1 and follower 2. To further investigate the game strategy choice of the two enterprises under the dynamic market environment changes, it is essential to compare the equilibrium profit of the enterprises implementing the cooperation strategy and those that do not. In other words, if $\Delta \pi_{i}=\pi_{i}^{C}-\pi_{i}^{D}\geq 0$ represents enterprise *i* is willing to cooperate, otherwise there is no willingness to cooperate. If and only if $\Delta \pi_{1}\geq 0\;\text{and}\;\Delta \pi_{2}\geq 0$, the leader and the follower will invest in data elements collaboratively.

**Fig 5:**
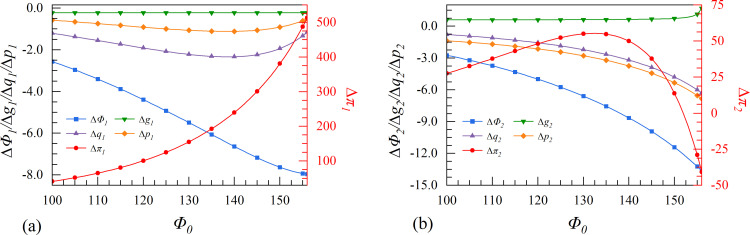
The effect of initial market size Φ_0_ on enterprises’ investment strategies.

**Fig 6:**
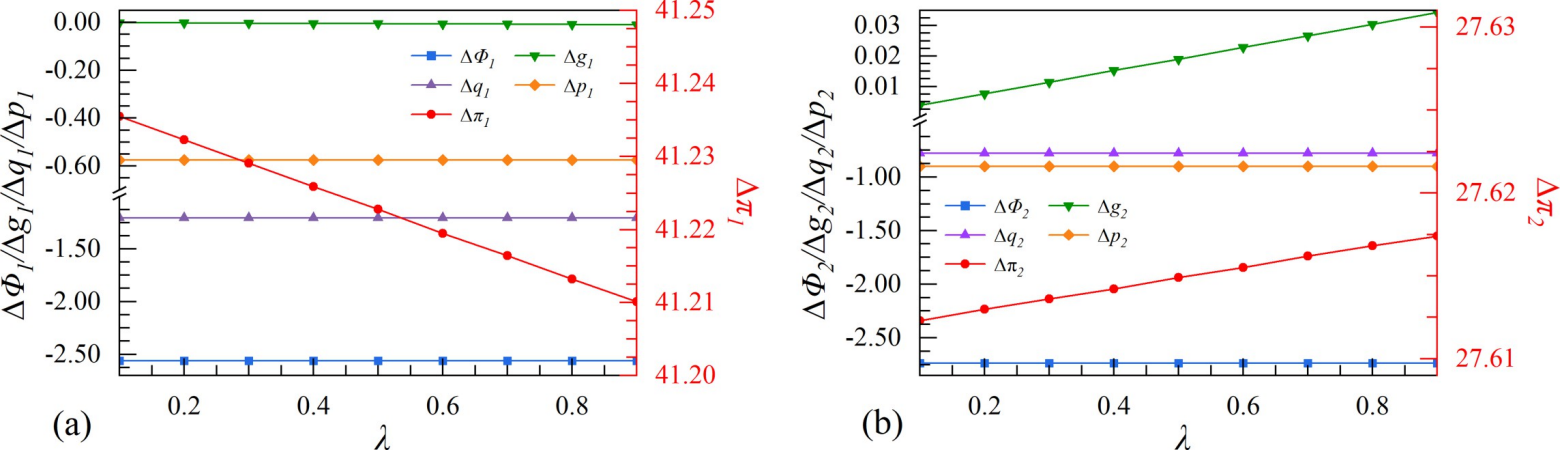
The effect of spillover coefficient λ on enterprises’ investment strategies.

**Fig 7:**
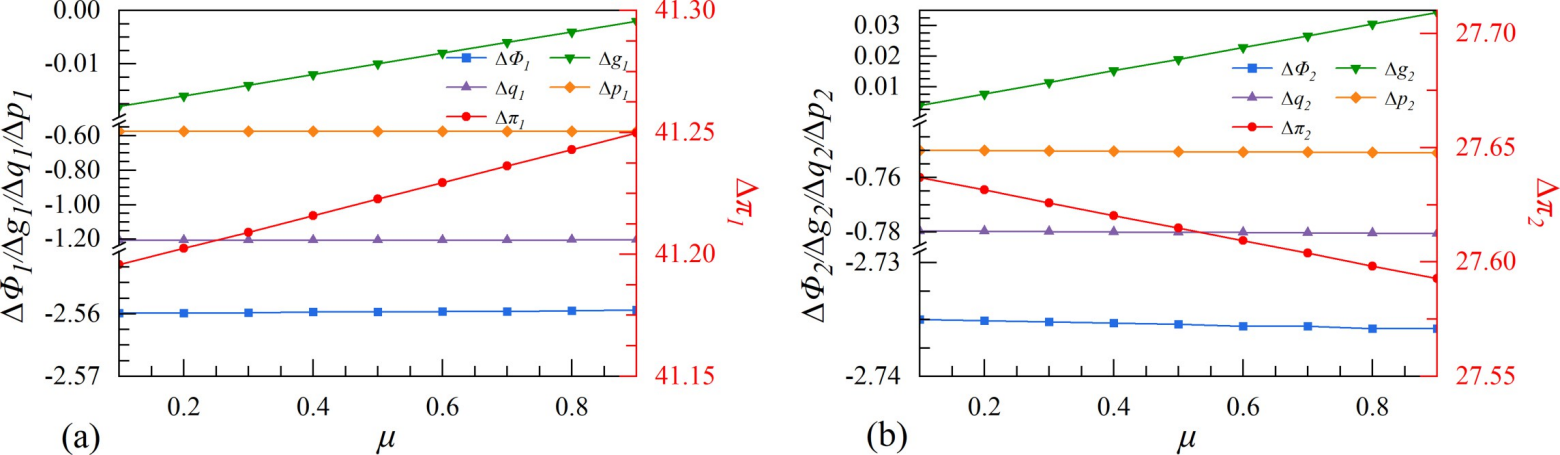
The effect of elasticity of substitution μ on enterprises’ investment strategies.

**Fig 8:**
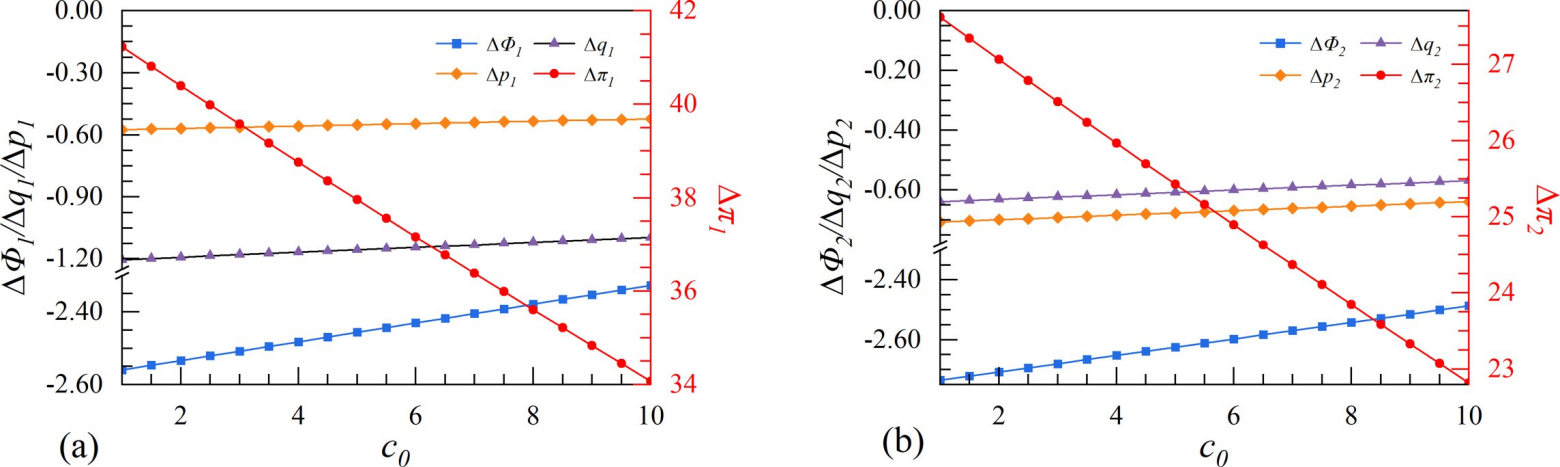
The effect of initial marginal production cost *c*_0_ on enterprises’ investment strategies.

**Fig 9:**
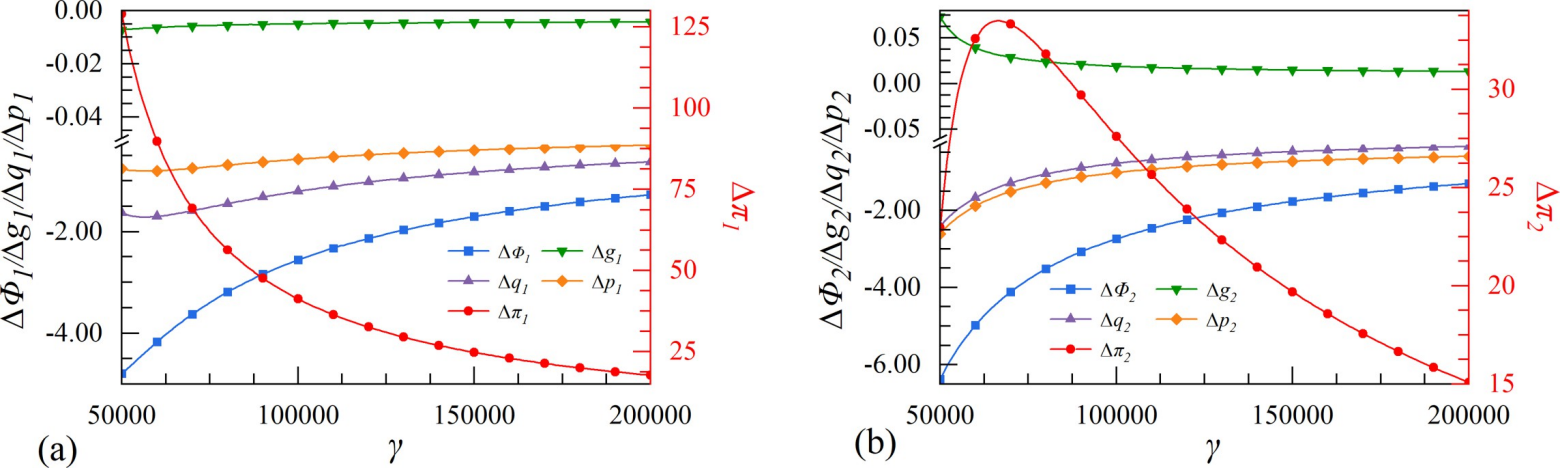
The effect of cost coefficient of digital assets γ on enterprises’ investment strategies.

**Fig 10:**
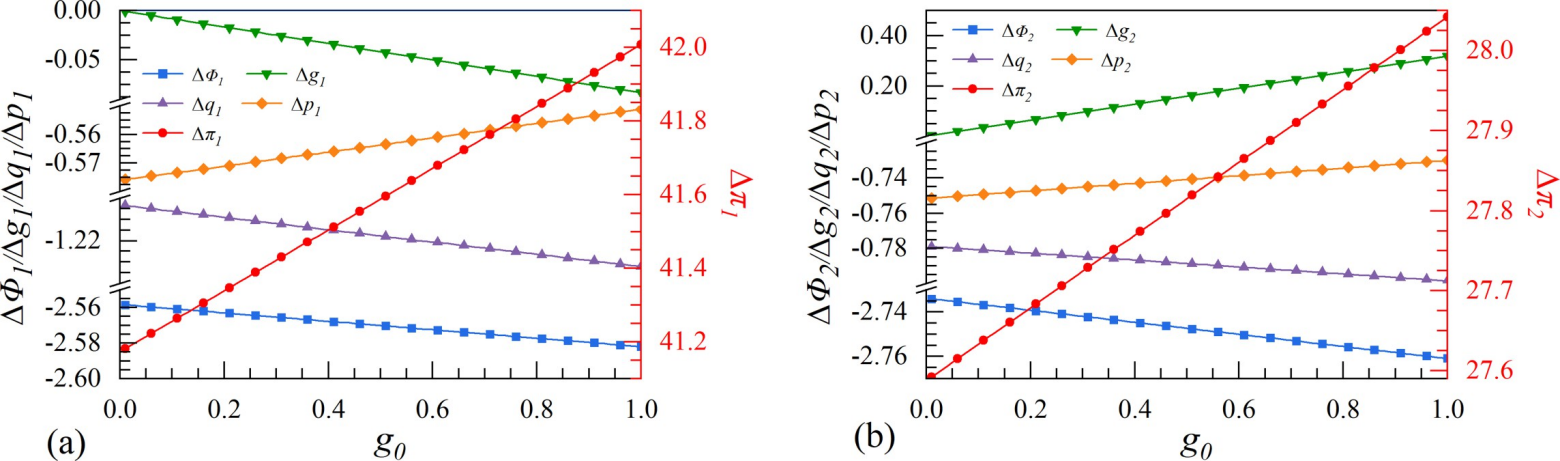
The effect of steady-state growth rate of economy *g*_0_ on enterprises’ investment strategies.

Based on the characteristics of the digital economy, this paper considers six scenarios for the change of market dynamic environment, as shown in Table 2. The variables are assigned values following the principles: first, by combining relevant research [62, 63] and considering the actual situation, such as the resource differences between leader and follower in the market; second, by interviewing and consulting with experts in the field of the system and executives of the company; and third, by making reasonable estimations and determining the initial parameters based on the model setup and simulation experiments. According to these principles, the values of parameters are set as follows: *Φ*_0_ = 100, λ = 0.5, *g*_0_ = 0.06, μ = 0.5, *c*_0_ = 1, γ = 100000. Especially, the effects of changes in enterprise profit and other factors from multiple market environments are presented and analyzed in detail from Figs 5–10.

**Table 2:** Description of parameter symbols.

Scenarios	Parameters	Description
I	Φ_0_	initial market size
II	λ	spillover coefficient
III	*g*_0_	steady-state growth rate of economy
IV	μ	elasticity of substitution
V	c_0_	initial marginal production cost
VI	γ	cost coefficient of digital assets

### 4.1. Scenarios I. Initial market size

Scenario I examines the characteristics of the initial market size. A larger market size indicates that the industry has reached saturation, resulting in higher market maturity and limited growth potential. From Fig 5, when the initial market size reaches a high level (Φ_0_ > 151), the follower has no incentive to invest in data elements collaboratively due to its negative relative profit. Meanwhile, the leader is always incentivized to participate in cooperation strategies. The results show that the cooperation strategy can only be implemented when the initial market size is not saturated.

The digital specialization of enterprises is driven by the expansion of the initial market size, which aims to improve productivity. In a saturated market, the Matthew effect, whereby the strongest enterprise becomes even stronger, has a significant impact on the profit of both enterprises. If two enterprises implement the cooperation strategy, the leader with greater influence and a more robust system will quickly capture residual market share. Therefore, the follower is not willing to engage in collaborative innovation when the initial market size is high (Φ_0_ > 151).

It is worth noting that the market size of the two enterprises increases significantly when they invest in data elements independently, compared to cooperating with others. This shows that the market expansion of enterprises may be constrained by cooperation strategy in mature markets. They tend to focus on customers whose objectives align with the cooperation strategy and conduct in-depth value analysis to uncover greater vertical value. Accordingly, as the market size shrinks, the output of the follower has been severely reduced. The digital economy operates on economies of scale, meaning that as the initial market size develops, the marginal production cost of enterprises decreases, leading to a drop in production cost. In particular, the cooperation strategy can complement the follower to further reduce product costs. Therefore, the cooperation strategy between both enterprises results in lower product prices. This trend becomes more pronounced as the initial market size increases.

### 4.2. Scenarios II. Spillover coefficient

Scenario II is based on the perspective of spillover effect in the digital economy. The increase in the spillover coefficient of an industry suggests that competitors can access technological advancements at a minimal cost. Therefore, enterprises must carefully consider the advantages and disadvantages when making strategic decisions regarding digital innovation. From Fig 6, both enterprises make more profit when they cooperate. However, with the increase of the spillover coefficient, the profit advantage of the leader in the cooperation strategy may be reduced, while the follower does the opposite.

By comparing the two strategies, it is observed that as the spillover increases, the growth rate of the follower’s data element continues to rise in the cooperation strategy. A high spillover coefficient allows the follower to benefit from advanced knowledge and technologies resulting from the digital innovation of the other party at minimal costs. In this case, the follower can partake in the leader’s copious knowledge spillovers, enhancing the growth rate of data element. Consequently, the profit of the follower will increase in the cooperation strategy, even if it may be potentially detrimental to the leader’s interests.

### 4.3. Scenarios III. Elasticity of substitution

Under scenario III, the elasticity of substitution represents the fungibility of two enterprises’ digital research results. A higher elasticity of substitution indicates a greater increase in the leading enterprise’s growth rate of data elements, which suggests significant advancements in digital innovation by the leader. On the contrary, if the elasticity of substitution is lower, then the effect of the leading enterprise’s investment in data elements is minimal, and it can be easily replaced by the digital research results of others. From Fig 7, the two enterprises will choose the collaborative innovation strategy regardless of the level of elasticity of substitution. As the elasticity of substitution increases, the leader will continue to benefit from choosing the cooperation strategy, while the follower will experience a weakened profit advantage from doing the same.

Fig 7 shows that as the elasticity of substitution increases, there are no significant differences in market size, output, or product price between the two enterprises that employ collaborative or independent innovation strategies. However, the growth rate of the follower’s digital assets is considerably higher in the collaborative strategy, and with the growth of the elasticity of substitution, which can compensate for the growth rate of the leader’s digital assets, is converging with the independent strategy. By integrating into the same innovation ecology, the two enterprises can more effectively solve the dilemma of research results’ susceptibility to change. This integration enhances both enterprises’ innovative capabilities considerably. Especially, the externality of the digital economy allows the following enterprise to share some of the digital research results in the cooperation strategy, which can optimize its weak digital foundation and low-structured digital ecology. The growth rate of the following enterprise’s digital assets experiences a significant increase following the replenishment of high elasticity of substitution.

### 4.4. Scenarios IV. Initial marginal production cost

The scenario IV examines the initial marginal production cost. The cost of an enterprise product consists of two components: the initial fixed cost and the marginal production cost. It is worth noting that high initial fixed costs may discourage enterprises from investing in data elements. According to Fig 8, the two enterprises will choose the cooperation strategy to mitigate the effect of high initial marginal production costs.

Enterprises can reduce the effect of high initial costs through the cooperation strategy, which enables them to transfer and share production factors between enterprises more efficiently. This cooperation facilitates optimization of resource allocation and leads to higher innovation performance. By employing the collaborative innovation strategy, the two enterprises can earn considerable profits, even though this profit margin may be compressed by the high initial cost.

The results indicate that high production costs significantly decrease the leader’s profit. Once the enterprises’ willingness to invest in digital innovation weakens, the possibility of product and service innovation is greatly reduced. Consequently, both enterprises produce similar products and services, causing them to become homogenized and lose competitive edge. As a result, the leader experiences greater market losses in innovation profit.

### 4.5. Scenarios V. Cost coefficient of digital assets

In scenario V, the increase in the cost coefficient of digital assets makes it difficult for the two enterprises to translate their investments in data elements into tangible results. In general, as the cost coefficient increases, enterprises become less willing to carry out digital innovation strategies. From Fig 9(B), when γ ϵ (50000,67000), the cooperation strategy is particularly advantageous for the follower. During the initial stage, when the cost coefficient of digital assets is low, the follower can collaborate with the leader to quickly learn and master new digital technologies and business models. This rapid learning and adaptability can help the follower improve its digital level in a short period and gain a competitive advantage. Besides, partnering with the leader allows the follower to share the costs and risks of digital transformation. This is particularly important as digital assets become increasingly challenging to transfer into tangible results. It helps the follower remain flexible and competitive, driving profit growth.

However, when γ > 67000, the excessive cost coefficient of digital assets impedes the growth of cooperation strategy on enterprise profit. Due to the inflated cost coefficient of digital assets, it is challenging for the follower to maintain its profit growth, causing its competitive advantage to gradually diminish and the profit growth rate to decline.

In general, collaborative innovation can serve as a means of supply and demand matching for two enterprises, thereby alleviating the cost pressure caused by the extremely low conversion rate of innovation income. Therefore, the cooperation strategy remains a better solution for the two enterprises.

### 4.6. Scenarios VI. Steady-state growth rate of economy

Scenario VI is based on the perspective of the steady-state growth rate of economy, which signifies the economic scale of this period. The economic system is highly coordinated with social resources and environmental capacity, resulting in a stable, healthy, and high-quality state of economic development. As the economy experiences consistent growth rates, digital enterprises will improve their service capabilities and adjust to a stable state that aligns with market size. According to Fig 10, both enterprises have chosen the cooperation strategy. It is worth noting that the growth rate of the follower’s data elements is expected to increase significantly.

In the high steady-state growth rate of economy, the leader and the follower who implement the collaborative innovation strategy will reap greater rewards in their innovation profits. Establishing a collaborative digital system between enterprises can enable them to gain a comprehensive understanding of the market, make informed decisions regarding the allocation of limited social resources, and develop digital innovations to address gaps in the market. Ultimately, this approach contributes to maintaining social and economic capital stability.

The comparison between the two strategies suggests that when the initial steady-state growth rate of economy increases, the growth rate of the follower’s digital assets increases as a result of the cooperation strategy, while that of the leader declines. This is because a society with a high steady-state growth rate of economy must utilize big data as technical support to realize innovation. Therefore, the follower can benefit greatly from the cooperation strategy, which accelerates the integration of technological progress and economic growth, ultimately achieving product innovation iteration.

## 5. The effect of data element strategies selection on industry and society

This section presents a simulation of the effect that the leader and the follower have on the overall industry innovation performance (*R*), consumer surplus (*V*), and societal wealth (*W*) under various scenarios of digital innovation strategies. This section presents the analysis following the principles outlined in the preceding section on parameter setting. We focus on three dynamic market environment characteristics: initial market size, initial marginal production cost, and cost coefficient of digital assets that have a major impact on the industry and society.

**Fig 11:**
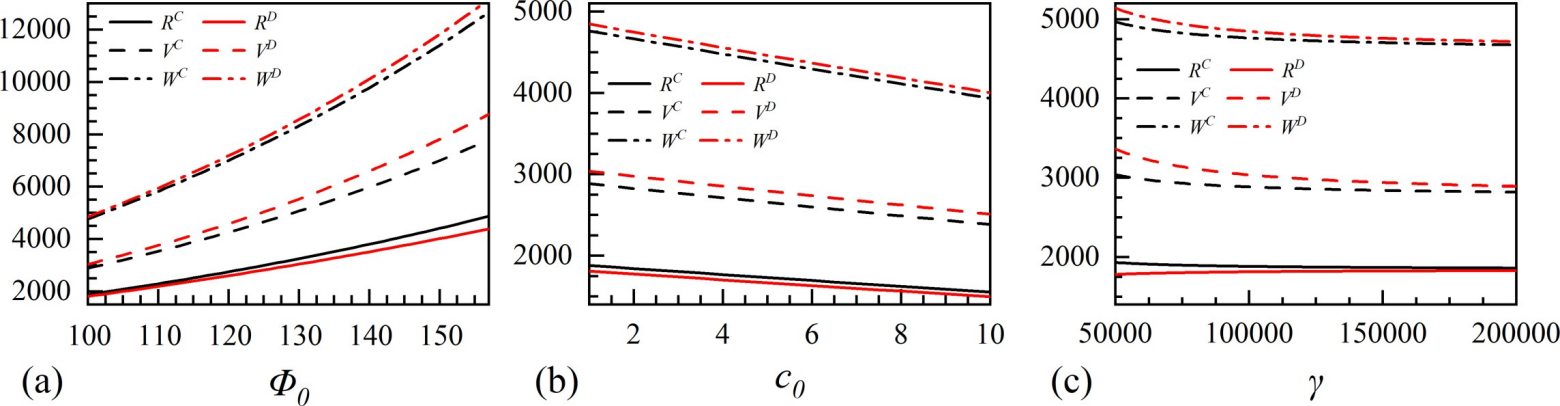
The effect of data element investment on industry and society.

Fig 11(A) shows that the overall industry innovation performance rises as the initial market size develops. Furthermore, the collaborative innovation strategy is more effective, and this difference becomes more pronounced as the initial market size grows. The initial market size development can drive enterprises to transform market states into high-value information and adjust their operation strategies rationally with rich digital resources as decision-making support. Consequently, the industry’s capacity for innovation remains high. Consumer surplus and social wealth increase as the initial market size grows, and attained via independent strategy is higher than that of collaborative strategy.

High initial marginal production costs can inhibit the development of the industry and society, as shown in Fig 11(B). The cooperation strategy can enhance an enterprise’s capacity to confront risks, adapt to complex environmental changes, and lead to higher industry performance. In addition, cooperation between enterprises can lead to resource complementarity and enhance market control, reducing unnecessary losses resulting from competition. When enterprises shift the cost burden to consumers, their desire to purchase products decreases, inhibiting the market’s consumption potential, which leads to a decrease in consumer surplus and social wealth.

Fig 11(C) illustrates that when the cost coefficient of digital assets is low (γ<125000), the industry can achieve a more substantial innovation performance through collaborative strategies. However, as the cost coefficient of digital assets increases, the two strategies exhibit no discernible difference. The high investment costs largely discourage enterprises from investing in data elements, resulting in a negligible difference in the impact of the two strategies at the industry and societal levels.

## 6. Discussion

In the contemporary business environment, organizations rely on data element projects as a means of facilitating decision-making and the formulation of plans. Nevertheless, research on the optimal strategies for digital innovation remains limited, making it challenging for organizations to effectively navigate the uncertainties of digital transformation. Previous studies have not adequately considered the impact of environmental factors that firms may face in digital transformation scenarios, nor have they considered the integrating perspective of the sequential game of firm output and the cooperative game mechanism based on overall profit maximization. Accordingly, this paper proposes a three-stage game model to construct the profit objective function under varying strategies and firms select output in a sequential game. Based on the model, the sensitivity analysis of environmental factors is conducted.

Our findings indicate that firms should prioritize the collaborative strategy for higher innovation profits, except for instances where the initial market size is excessive. It is therefore of interest to ascertain whether some alternative motives or mechanisms could influence this strategic decision. Meanwhile, the cooperative strategy is beneficial for followers who wish to accelerate the construction of digital assets and enhance digital service capabilities. However, the collaborative strategy constrains the market size of both firms.

The research concerns two enterprises undergoing digital transformation. However, in reality, the co-opetition relations between multiple enterprises and other subjects are common. Consequently, the next stage of research can be expanded to more complex game scenarios. Besides, the model could be enriched by incorporating organizational flexibility and production efficiency to represent the inequality of market power, which would lead to valuable conclusions. Research on digital collaboration decision-making in complex market environments necessitates the integration of diverse research methods and approaches. In this study, the primary method for identifying factors of cooperation was through sensitivity analysis, which inevitably resulted in insufficient observation of firms’ decision-making behaviors under long-term trends. In the future, the system dynamics method that focuses on the evolution of different collaboration strategies over the long term may prove a meaningful avenue of inquiry, providing more suggestions for business management and decision-making.

## 7. Conclusions

### 7.1. Main findings

As digital transformation accelerates and data resources become more valuable, this study focuses on the choice of digital innovation paths for enterprises with different market power in dynamic market environments. Additionally, the analysis considers the influence of different strategies on the industry and society.

The conclusions are drawn as follows:

1. In most scenarios, the cooperation strategy can lead to higher innovation performance for both leader and follower compared to investing in data elements independently. However, in mature industries, cooperation may not align with the interests of the follower, who may choose to invest in data elements independently.
2. In any market environment, the collaborative innovation strategy can lead to a superior digital assets growth rate in the following enterprises. Additionally, by binding enterprises into an interest group, the cooperation strategy may cause them to lose their original market scale. However, the two enterprises can share market resources, realize economies of scale from the perspective of enterprise cooperation as a whole, and reduce the price of products through the cooperation strategy.
3. The industry is capable of attaining enhanced innovation performance through the implementation of cooperative strategies. Conversely, independent strategies tend to result in higher social welfare and consumer surplus. The disparity between the two strategies may be eroded by excessive cost coefficients and magnified by mature market.

### 7.2. Implications

This paper examines the digital innovation strategies of leaders and followers in various dynamic market environments. It guides enterprises interested in participating in digital transformation on how to adapt decision-making behaviors in response to environmental changes. Generally, we provide several suggestions for enterprises to enhance digital innovation:

First, it is crucial for enterprises to actively participate in collaborative development in the era of the digital economy. Leading enterprises in mature industries benefit from the substantial market size by efficiently incorporating digital elements. Influenced by the Matthew effect, the adoption of the collaborative innovation strategy conflicts with the interests of followers. Cooperation among enterprises is feasible in emerging industries, where the market size is in its nascent stage. Second, followers generally face constraints due to low digital proficiency and limited resources. To mitigate the impact of competitors, it is necessary to accelerate the digital transformation process. Therefore, followers engage in digital cooperation projects to strengthen their data element application capability, which has the potential to transform data-driven performance. Third, excessively high initial production costs serve to impede the advancement of both the industry and society. Intervention is an effective means of preventing the negative impact. At this time, it is appropriate for the government to take intervention through policy combinations across multiple fields [64]. These policies could include increasing subsidies for innovation capital and promoting additional tax and fee reductions for industrial research and development through financial policy tools.

Making appropriate strategies is crucial for the digital transformation path of enterprises in data-driven innovation [65]. This study analyzes the data elements and their dependency relations in data-driven innovation from game theory. Further, we incorporate digital elements as a means of gaining advantages in the competitive industrial innovation performance mechanism [66] and suggest various types of enterprises in selecting suitable strategies. For intricate circumstances, enterprises should choose large-scale supply chain partners that can adapt to future challenges and then disseminate the value added to data elements [67]. Although this paper does not provide a direct conclusion on the aforementioned issues, it suggests that complex problems can be broken down into simpler ones through system reductionism. Therefore, the analytical framework presented in this paper can be expanded to address intricate game scenarios.

## Supporting information

S1 Dataset (XLSX)
